# Spatiotemporal Variability and Sound Characterization in Silver Croaker *Plagioscion squamosissimus* (Sciaenidae) in the Central Amazon

**DOI:** 10.1371/journal.pone.0099326

**Published:** 2014-08-06

**Authors:** Alfredo Borie, Hin-Kiu Mok, Ning L. Chao, Michael L. Fine

**Affiliations:** 1 Department of Fishery, Amazon Federal University, Manaus, Brazil; 2 Department of Oceanography and Asia-Pacific Ocean Research Center, National Sun Yat-sen University, Kaoshiung, Taiwan; 3 National Museum of Marine Biology and Aquarium, Checheng, Pingtung, Taiwan; 4 Department of Biology, Virginia Commonwealth University, Richmond, Virginia, United States of America; Claremont Colleges, United States of America

## Abstract

**Background:**

The fish family Sciaenidae has numerous species that produce sounds with superfast muscles that vibrate the swimbladder. These muscles form post embryonically and undergo seasonal hypertrophy-atrophy cycles. The family has been the focus of numerous passive acoustic studies to localize spatial and temporal occurrence of spawning aggregations. Fishes produce disturbance calls when hand-held, and males form aggregations in late afternoon and produce advertisement calls to attract females for mating. Previous studies on five continents have been confined to temperate species. Here we examine the calls of the silver croaker *Plagioscion squamosissimus*, a freshwater equatorial species, which experiences constant photoperiod, minimal temperature variation but seasonal changes in water depth and color, pH and conductivity.

**Methods and Principal Findings:**

Dissections indicate that sonic muscles are present exclusively in males and that muscles are thicker and redder during the mating season. Disturbance calls were recorded in hand-held fish during the low-water mating season and high-water period outside of the mating season. Advertisement calls were recorded from wild fish that formed aggregations in both periods but only during the mating season from fish in large cages. Disturbance calls consist of a series of short individual pulses in mature males. Advertisement calls start with single and paired pulses followed by greater amplitude multi-pulse bursts with higher peak frequencies than in disturbance calls. Advertisement-like calls also occur in aggregations during the off season, but bursts are shorter with fewer pulses.

**Conclusions and Significance:**

Silver croaker produce complex advertisement calls that vary in amplitude, number of cycles per burst and burst duration of their calls. Unlike temperate sciaenids, which only call during the spawning season, silver croaker produce advertisement calls in both seasons. Sonic muscles are thinner, and bursts are shorter than at the spawning peak, but males still produce complex calls outside of the mating season.

## Introduction

Sciaenid fishes, with common names such as croakers and drums, produce advertisement calls during the reproductive season [1], [2], [3], [4], [5], [6]. The sounds are produced by contraction of a pair of sonic muscles typically present in males or occasionally in both sexes [7]. Sonic muscles may be extrinsic and run from an aponeurosis on the dorsal bladder to a tendon on the ventral midline [8] or intrinsic and confined to the swimbladder walls as in black drum [9], [10]. The sonic muscles form during puberty when the juvenile gonads differentiate into testes [11] and in weakfish have the classic morphology of superfast fish sonic muscles with a core of sarcoplasm surrounded by a radially-arranged contractile cylinder consisting of alternating ribbons of sarcoplasmic reticulum and myofibrils [12]. Under androgenic control [13], the sonic muscles progress through a yearly hypertrophy-atrophy cycle in which the muscles increase in mass, thickness and fiber diameter to a peak for the mating season and atrophy afterward [14]. Muscle protein, glycogen and lipids also increase seasonally [14].

Using passive-acoustics, fishery biologists have mapped the distribution of vocalizing aggregations of numerous soniferous sciaenids, e.g., *Bairdiella chrysoura*, *Cynoscion nebulosus*, *Pogonius cromis* [10], [15], *Protonibea diacanthus* [4], *Micropogonias furnieri* [16], *Cynoscion regalis* [1], to name a few. Passive-acoustics is a non-visual, non-invasive and non-destructive tool that provides information about the distribution, daily and seasonal activities patterns of fishes [17].

South American freshwater sciaenids are represented by four genera: *Pachypops*, *Pachyuru*, *Plagioscion*, and *Petilipinni* [18]. *Plagioscion* contains about 15 species [19], which occur in the major river drainages to the Caribbean Sea and Atlantic (from the Magdalena River in Colombia to La Plata on the border between Argentina and Uruguay).

The silver croaker *Plagioscion squamosissimus* is a benthopelagic species inhabiting the margins of lakes and mid-Amazon rivers [20], where habitats are characterized by water color: white, clear, black and mixed rivers. In caged fish sexual maturation occurs in October and November and January and February during low and rising water levels, with predominance of black water. Both conductivity and pH changes are potential triggers for gonadal maturation [21].

Little is known about the distribution of their spawning grounds. The goals of this study are to characterize the acoustic signals of silver croaker and qualitative development of sonic muscles at different times of the year and to survey the spatial and temporal distribution of their calls in the confluence of the Rivers Negro and Solimões. We have recorded underwater sounds in this area produced by catfishes, characids and pink dolphin, all of which have different acoustic signatures.

## Materials and Methods

### Ethics statement

No specific permission was required to record fish sounds from the River Negro, River Solimões or Lake Catalão, and *Plagioscion squamosissimus* is not a protected species.

Animals were not collected for wild recordings, which were totally noninvasive, i.e. a hydrophone was placed in the water. Fish in cages were collected under “*Licença permanente para coleta de material zoológico*” Número: 12273-1 to Ning Labbish Chao from the Brazilian Environmental Institution (IBAMA).

The Federal Amazon University is in the process of forming an IACUC, which did not exist when this work was done. However, the research was approved by a graduate research committee, and no animals were harmed: they were recorded underwater or were briefly hand-held and then returned to their cages.

### Study area

**Figure 1:**
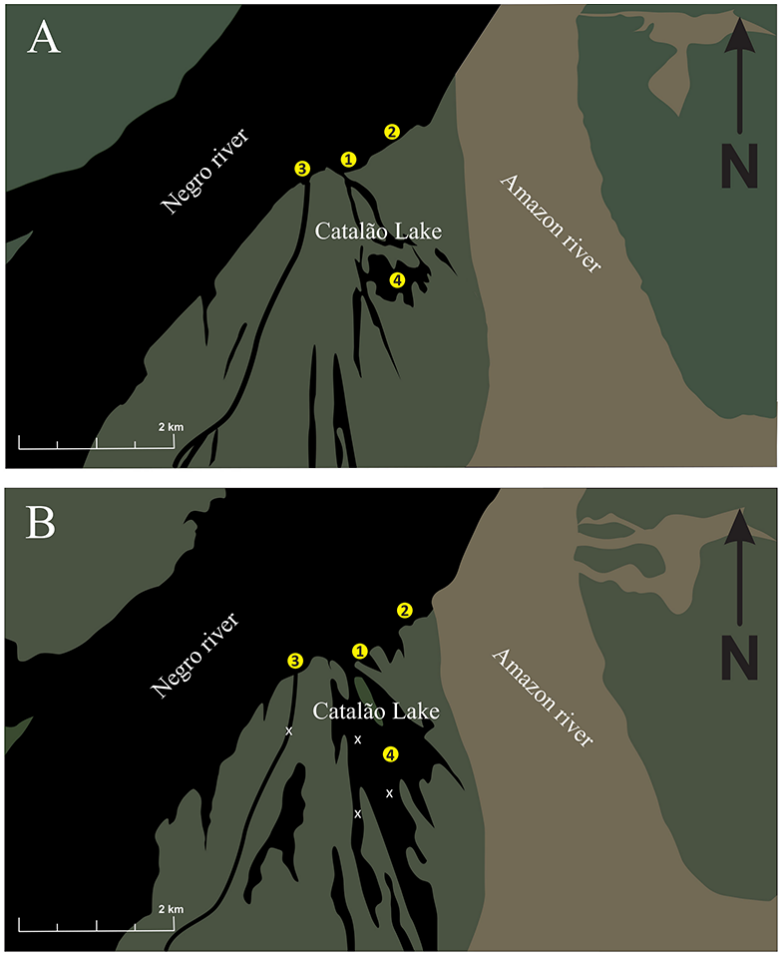
Study area. The hand-held disturbance and cage calls where recorded in a floating house laboratory (2). Wild calls were recorded from Xiboreno Channel (1); Catalão Island (3) and Catalão Lake (4). Xs indicate sites negative for sound production. A. Low-water season. B. High-water season.

Underwater recordings were made in the mouth of Negro River, the main black water Amazon River tributary (Fig. 1) and in Catalão Lake, which connects to the Solimões River during the high water season (May–July) and rarely disconnects completely from the River Negro in the dry season. The water characteristics of this area vary seasonally with fluctuating water levels; the water is predominantly black during low waters and white in high waters influenced by white water of the Solimões River. White water rivers, like the Solimões have a muddy color, an alkaline to neutral pH, and a high sediment load originating from the Andes [22], [23]. Black water Rivers like Negro are black due to a high concentration of dissolved organic carbon, have lower suspended sediment, and are usually acidic [24].

### Sound recording

Fish sounds were recorded with an H2a hydrophone (Aquarian Audio), with frequency range of 10 Hz-100 kHz connected to a digital recorder Olympus WS-400S (WMA format and sampling frequency 44.1 kHz). We recorded voluntary calls from captive fish in cages and from fish in the wild during low-water (November to December, 2009), and high-water seasons (June to July, 2010). Hand-held disturbance calls in air were recorded with an internal microphone in the recorder; captive and wild fish were recorded in late afternoon (ca 05∶00 pm).

### Hand-held disturbance calls

Fish were recorded during the mating season (low waters) from 10 individuals (mean 240±20 mm standard length, SL) maintained in cages as breeding stock and outside of the mating season from 9 individuals (mean 210±30 mm SL) caught near the mouth of Catalão Lake in high waters (Site 2, Fig. 1). Each fish was hand-held for 1 to 2 min with the recorder placed 1 or 2 cm from its abdominal area.

### Voluntary calls from cages

Sounds were recorded from a large but unknown number of fish (range 200–340 mm SL) in three 12 m^3^ (2×3×2) cages, each stocked at a density of approximately four fish/m^3^. Males and females were held together for one year at the right margin of Negro River (Site 2, Fig. 1). Sex was determined by abdominal massage and cannula extraction. A hydrophone was submerged to 1 m for 15–60 min, and recordings were made at dusk.

### Voluntary calls from the wild

Fish were recorded at the mouth of Negro River during low- and high-water season and in the Catalão Lake in high-waters (Fig. 1). Initially we explored numerous potential sites including inside of Xiboreno Channel and several places in Catalão Lake that were negative or contained low-amplitude sounds indicative of far-away calls.

Fish were recorded monthly during three days in each site at the mouth of Xiboreno Channel (Site 1; Fig. 1), the mouth of Negro River (right margin, Site 3; Fig. 1) and Catalão Lake (Site 4; Fig. 1). Hydrophone recordings were made at 1 to 5 m for 30–120 min day^−1^. Water depth was measured to the nearest meter with an echo sounder.

### Signal analysis

Calls were edited with Easy Audio Cutter software and analyzed with Raven Pro v1.4. We measured, number of pulses per burst, burst duration, pulse duration, inter-pulse interval, pulse period, pulse rate, period of the greatest amplitude cycle within a pulse, peak frequency, low and high frequency. Power spectra employed a 3,000 point Hanning window with 50% overlap (bandwidth 10.8 Hz). One-way ANOVA followed by Turkey’s test was used to compare call parameters across recordings. We also measured the interval between the final pulse in the pulse series and the first pulse in the burst (n = 17) in four recordings to indicate the close association of pulses and bursts.

## Results

### Seasonality and Distribution of Callers

Disturbance calls were recorded from hand-held males during the low-water mating season and during the high-water period outside of the mating season. However, spontaneous sounds from caged individuals were present only during the low-water mating season. Sounds from wild individuals occurred in choruses in both seasons. In the natural habitat wild calls (n = 127) from putative mating aggregations were recorded at depths between 8 and 12 m north of the Catalão Island and occasionally from Xiboreno channel (Sites 3 and 1, respectively in Fig. 1). No calls were recorded in other parts of Catalão Lake during the same period despite hydrophone surveys of the south shore. Low amplitude sounds were recorded in shallow water (3 m) sites in southern and western Catalão Lake, and sounds were absent inside the Xiboreno Channel (Fig. 1). During the high-water non-spawning season, calls were again restricted to specific areas in Catalão Lake (Sites 4 in Fig. 1) at depths of about 20 m. Therefore, fish formed aggregations in restricted locations in both seasons in late afternoons.

**Figure 2:**
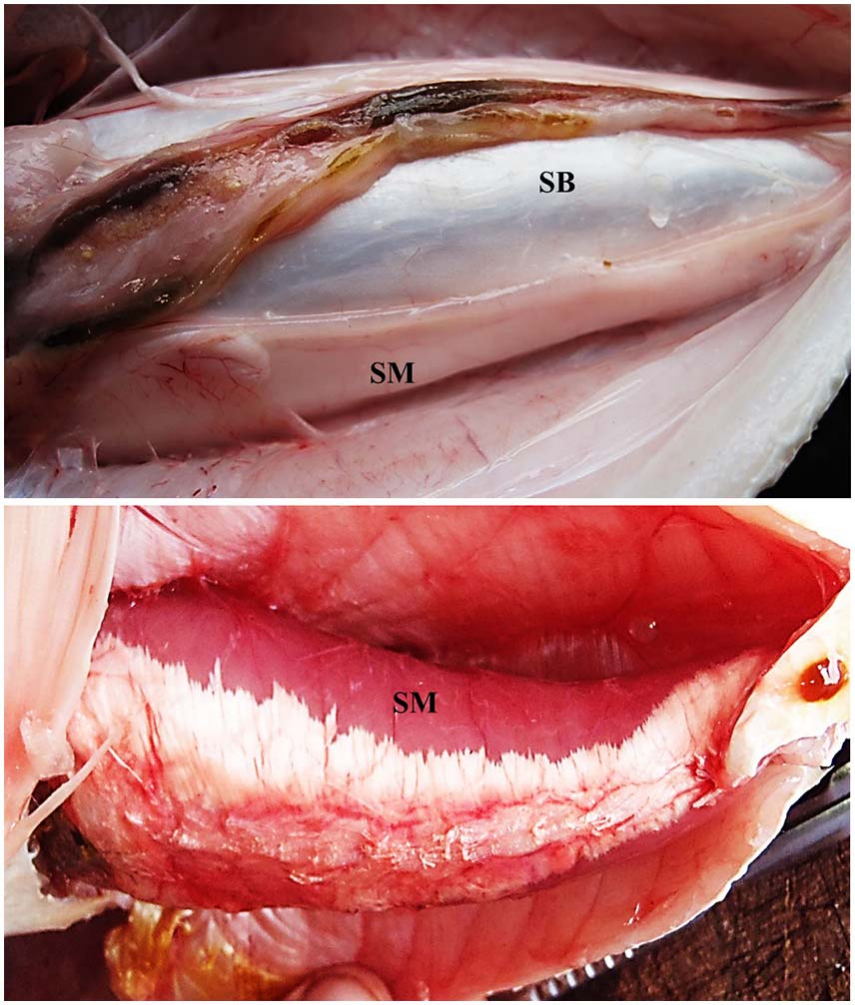
Photographs of Sonic muscle of *Plagioscion squamosissimus*. (A) Out of the mating season; note sonic muscles are translucent and thin. (B) Thicker muscle with a red color during the mating season. SwB: Swimbladder, Tes: Testes.

Dissections of 18 males and 22 females indicated sonic muscles were present only in males. Muscles increased in thickness and assumed a dark-red color during the mating season (Fig. 2).

### Sound production

**Figure 3:**
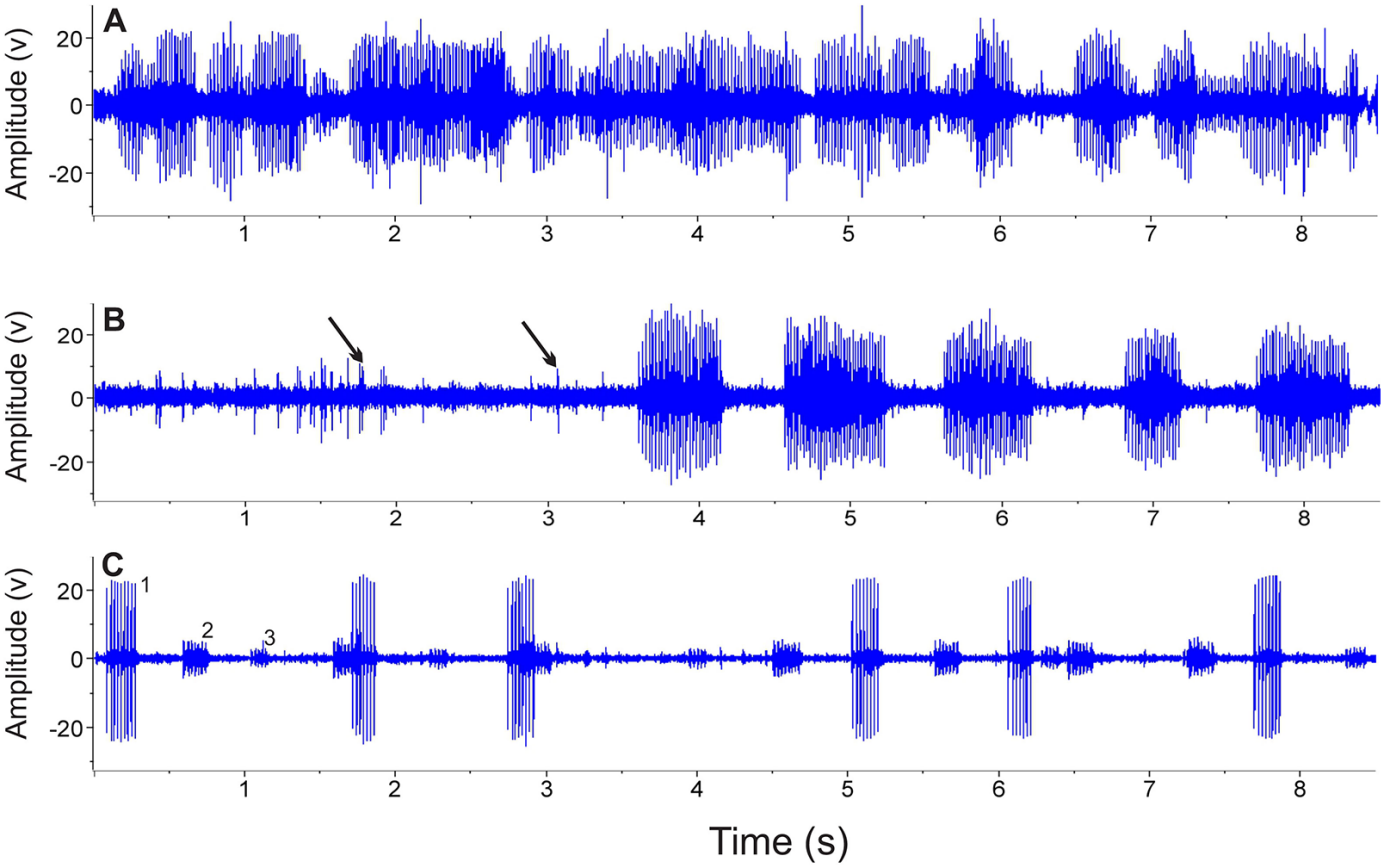
Oscillograms of advertisement calls of *Plagioscion squamosissimus*. (A) dense chorus recorder during the low-water mating season. (B) recording from an individual fish during the mating season, with double and single pulses (arrows) occurring before a series of bursts. (C) chorus from three fish in the high-water season (1, 2, 3) at various distances from the hydrophone. Note the busts are shorter and further apart than in (B).

**Figure 4:**
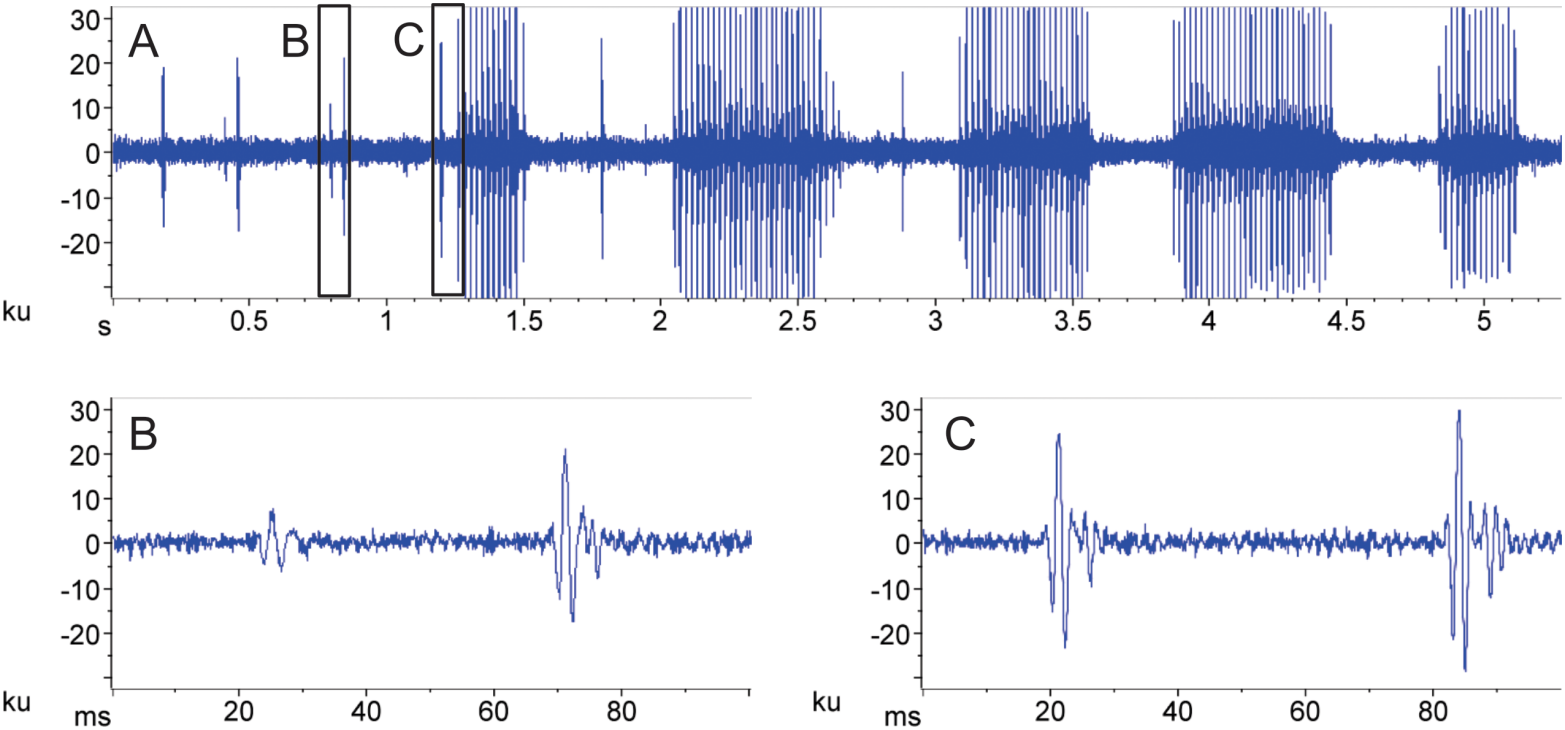
Complex sounds of *Plagioscion squamosissimus*. (A) Call from one individual showing the single and double pulses preceding a burst. (B) expanded double pulse. (C) last individual pulse and the first pulse of a burst. Note the similarity in waveforms.

Disturbance calls were audible in air from handled fish>19.0 cm SL. However, individuals between 13–19 cm SL produced vibrations felt by hand but that did not produce audible sound. Calls produced in nature and in cages resulted in dense choruses that obscured sounds from individual fish (Fig. 3a). Although difficult it was possible in some cases to identify bursts that likely come from nearby individuals. Occasionally, advertisement calls from individuals were recorded, and they had a complex structure that started with several single or double pulses that varied in amplitude before a series of higher amplitude rapid bursts, which sometimes were interspersed with single pulses (Fig. 3b, 4a and b). Furthermore, bursts were always paired with pulses (Fig. 4a and c) so that it is unlikely they were produced by multiple individuals or by other species. Measurements of 17 calls from four individuals with particularly clear waveforms indicated that the interval between the last single pulse and the first pulse of the burst averaged 162±30 ms (range 46 to 573 ms). Parsimony suggests the close temporal relationship between pulses and bursts indicates they were likely produced by the same individual. Therefore recordings indicate that the fish can vary the amplitude and temporal properties of signals. Choruses were also present during high water, and these typically had fewer callers allowing individuals to be distinguished routinely (Fig. 3c). See Files S1, S2 and S3 for recordings of choruses.

**Figure 5:**
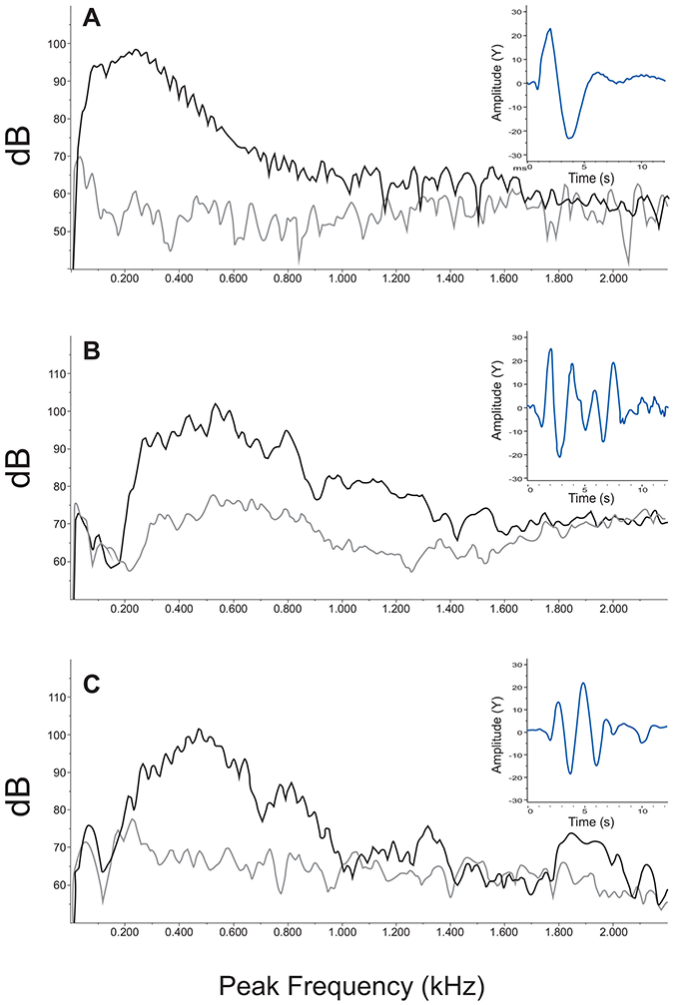
Frequency spectra of calls, background soundscape (inferior line) and pulse waveform (insets) from *Plagioscion squamosissimus*. (A) disturbance call. (B) advertisement call from the low-water mating season. (C) advertisement call from outside the mating season (high-water), Hanning 3000 points with 50% overlap (bandwidth 10.8 Hz).

During both seasons the pulse waveform tended to have a couple of high-amplitude cycles before several cycles of weaker amplitude (Fig. 5b and c insets).

The power spectrum of disturbance calls had a wide frequency distribution that increased approximately 30 dB to a peak at about 225 Hz, and then decreased about 30 dB to about 700 Hz, after which amplitude decreased more slowly to about 1000 Hz and contacted the noise floor. However, much of the signal continued above the noise levels until about 1800 Hz. An interesting feature of the spectrum in both aerial and underwater calls was a series of small peaks at regular intervals (ca 40 Hz) that could be as much as 3 dB above the baseline signal level (Fig. 5a). These peaks occurred variably in different calls but were present in all selections in air and underwater. These peaks did not correlate in any obvious way to the period of the signal. Peaks in underwater sounds occurred at similar frequency intervals (Fig. 5b and c).

The underwater spectrum of the advertisement call from the low-water mating season increased about 40 dB from about 200 Hz to a peak frequency at 500 Hz. The peak frequency therefore was almost twice as high as in the disturbance call. As visible in the insets, the time to complete a sound cycle is shorter for the advertisement than the disturbance calls (Fig. 5 inset). The high-amplitude portion of the spectrum continued to higher frequencies (ca 900 Hz) than in the disturbance calls. Energy in the call continued above the noise level to about 2000 Hz. Spectra of calls recorded outside of the mating season were relatively similar to that of the advertisement call although there was less energy at higher frequencies and noise contacted background at about 1000 Hz. The underwater sound spectrum during low water peaked between 500 and 600 Hz, although the water was calm with no riffles or waterfalls. High-pass filtering the call at 500 Hz and listening indicated a continuous white-noise type “shhh” sound that likely reflects biological activity but was not close enough to pick out individual features of sound-producing animals Fig. 5b). In fact the noise spectrum at mid-frequencies resembled the call spectrum suggesting that choruses of silver croaker are a major contributor to the underwater soundscape. This mid-frequency feature of the spectrum was not present outside of the mating season (Fig. 5c) suggesting less calling activity.

### Quantitative Seasonal Differences

**Figure 6:**
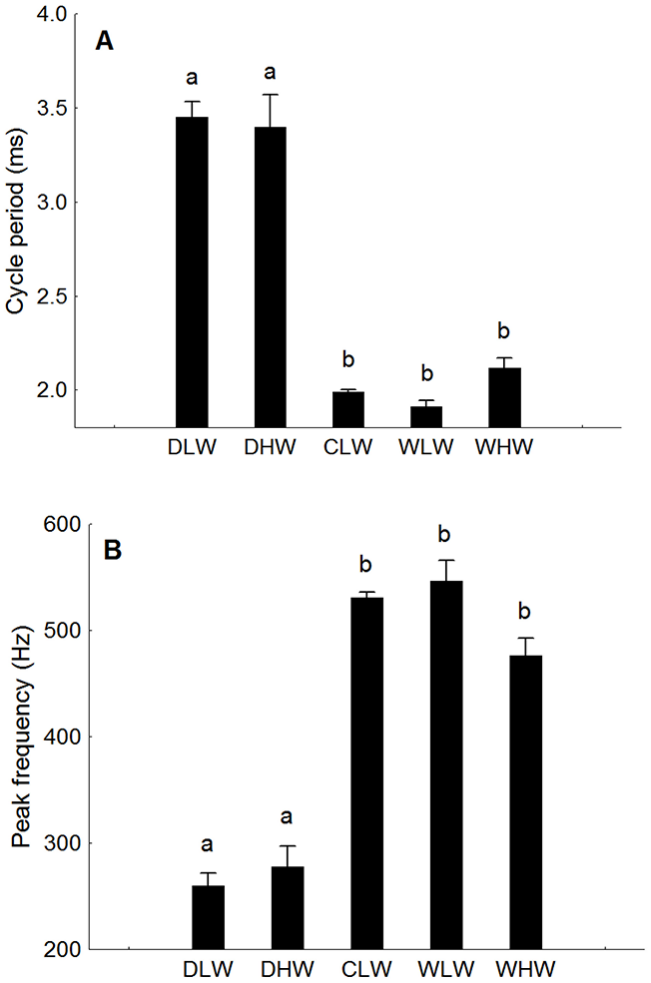
Longer period cycles generate lower peak frequencies in *Plagioscion squamosissimus*. (A) cycle period of high amplitude pulse and (B) peak frequency. DLW: disturbance call low-water, DHW: disturbance high-water, CLW: cage low-water, WLW: wild low-water, WLW: wild high-water. Different letters indicate significant differences (p<0.01).

**Figure 7:**
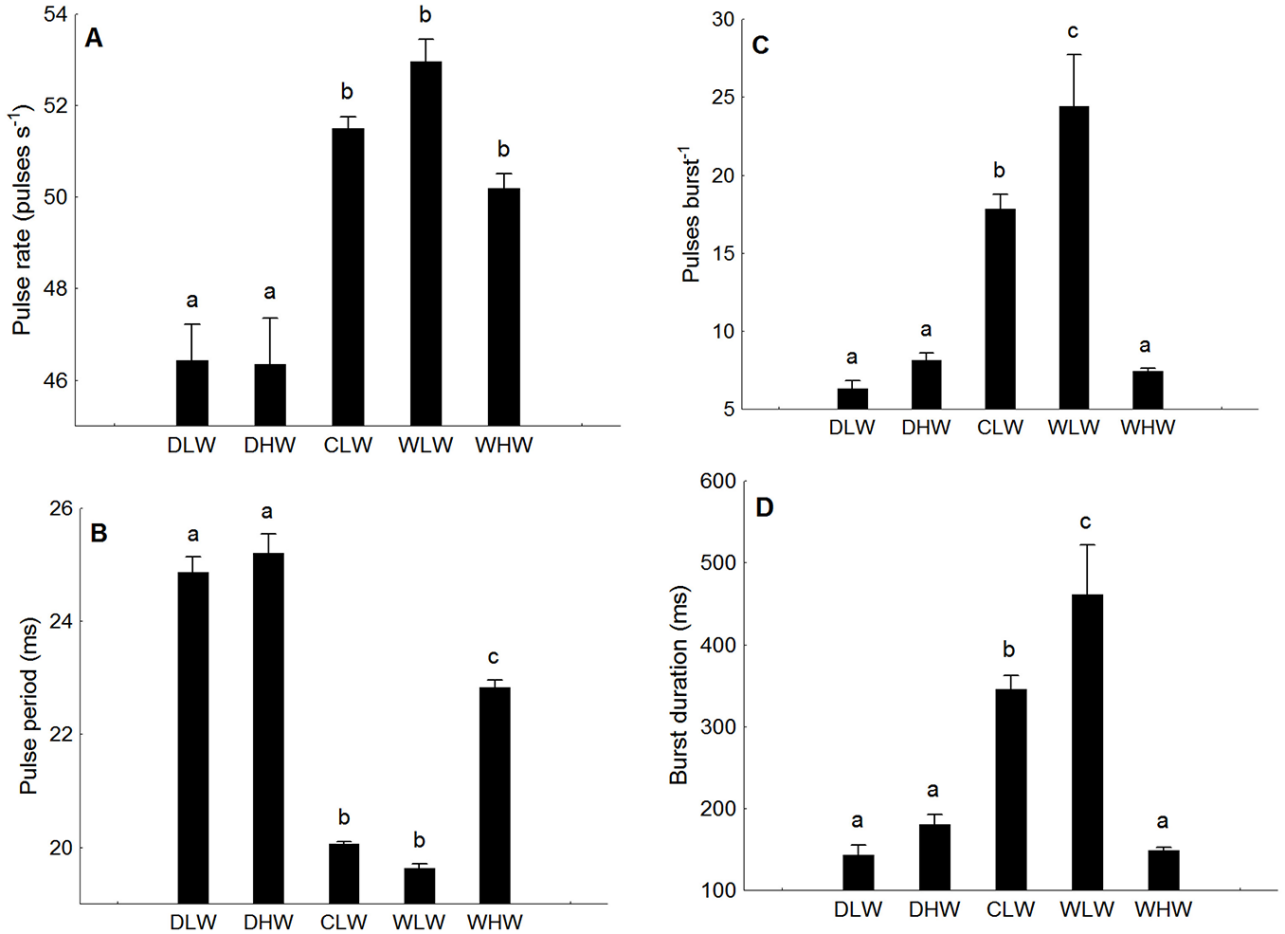
Mean + SE of temporal parameters of calls from *Plagioscion squamosissimus*. (A) pulse rate, (B) pulse period, (C) pulse per burst, (D) burst duration for a calls of *Plagioscion squamosissimus*. DLW: disturbance call low-water, DHW: disturbance high-water, CLW: Cage low-water, WLW: wild low-water, WLW: wild high-water. Different letters indicate significant differences (p<0.01).

A number of parameters changed across recordings, in some cases seasonally and some with recording situation (Table 1). For most parameters in low-water cage values were similar to wild recordings. Period of the most intense cycle decreased from about 3.5 ms for disturbance calls in both seasons to about 2.0 ms for cage and wild calls indicative of extremely fast muscle (Fig. 6a). The shorter period explains the higher peak frequency of advertisement calls, centering around 500 Hz for caged and wild individuals and less than 260 Hz for disturbance calls (Fig. 6b). Pulses per burst and burst duration, which obviously correlate, were lower in disturbance and high-water calls than in low-water calls from the mating season in both wild and cage calls (Fig. 7). Pulse rate was lowest in disturbance calls and higher in cage and wild calls from both seasons. For pulse period however, pulses occurred at ca 25 ms intervals for disturbance calls, and about 20 ms for low-water wild and cage calls. Wild calls from high water were intermediate between low-water and disturbance calls. In summary low-water calls during the mating season had longer bursts with more pulses and shorter intervals between pulses than high water calls. See Table 1 for ANOVA statistics.

**Table 1:** Mean ± SE and range in parenthesis of various parameters recorded from *Plagioscion squamosissimus* under different conditions.

	DLW	DHW	CLW	WLW	WHW	F	df	p
n of recordings	10	9	10	8	10			
n of burst	68	36	107	29	68			
Pulses burst^−1^	6.3±0.5^a^ (2–22)	8.1±0.5^a^ (3–14)	17.8±0.9^b^ (3–62)	24.4±3.3^c^ (9–89)	7.4±0.2^a^ (4–11)	45.8	4,303	<0.001
Burst duration (ms)	143±12^a^ (32–486)	180±12^a^ (63–342)	345±17^b^ (59–1207)	461±61^c^ (158–1622)	148±4^a^ (75–220)	40.3	4,303	<0.001
Pulse duration (ms)	10.1±0.1^a^ (7–14)	6.6±0.1^b^ (3–18)	9.1±0.0^c^ (5–17)	9.1±0.1^c^ (4–16)	7.5±0.1^d^ (4–13)	265.7	4,384	<0.001
Inter-pulse interval (ms)	14.8±0.3^a^ (6–45)	18.5±0.3^c^ (7–38)	10.9±0.1^b^ (3–26)	10.5±0.1^b^ (5–18)	15.2±0.1^a^ (7–25)	579.2	4,353	<0.001
Pulse period (ms)	24.9±0.3^a^ (8–32)	25.1±0.3^a^ (5–45)	20.0±0.0^b^ (8–35)	19.6±0.1^b^ (15–29)	22.7±0.1^c^ (9–32)	433.2	4, 354	<0.001
Pulse rate (pulses s^−1^)	46.4±0.7^a^ (32–63)	46.3±1.0^a^ (37–63)	51.4±0.2^b^ (45–63)	52.9±0.4^b^ (47–57)	50.1±0.3^b^ (45–55)	24.9	4,303	<0.001
Pulse cycle (ms)	3.4±0.1^a^ (2–5)	3.3±0.0^a^ (2–6)	1.9±0.0^b^ (1–2)	1.9±0.0^b^ (1–2)	2.1±0.1^b^ (1–3)	102.2	4,361	<0.001
Peak frequency (Hz)	260±127^a^ (172–775)	278±20^a^ (172–517)	530±6^b^ (345–689)	547±19^b^ (345–861)	476±16^b^ (172–689)	112.7	4,303	<0.001
Low frequency (Hz)	87±4^a^ (50–215)	122±8^a^ (54–303)	246±6^b^ (108–377)	166±9^c^ (68–306)	185±7^c^ (69–353)	96.6	4,303	<0.001
High frequency (Hz)	967±40^a^ (574–2401)	1019±33^a^ (511–1488)	1427±33^b^ (943–2828)	15525±41^b^ (987–1908)	1152±25^a^ (442–1693)	39.9	4,303	<0.001

DLW: disturbance call low-water, DHW: disturbance high-water, CLW: Cage low-water, WLW: wild low-water and WLW: wild high-water. Different superscript letters indicate means that are significantly different.

## Discussion

Acoustic monitoring indicates that *Plagioscion squamosissimus* calls from spawning choruses in restricted locations. Surveys from a number of Amazon rivers indicate that sites where rivers converge are hot spots for sound production and mating activity, and there is generally less calling up or downstream from such sites (Borie unpublished data).

In temperate latitudes males of a number of sciaenid species produce advertisement calls in spawning aggregations during the mating season (see introduction for references). A seasonal study of weakfish indicates they cease calling outside of the mating season [1]. This study on *Plagioscion squamosissimus* in the Amazon is the first report of seasonal changes in sound parameters in a fish from the tropics. *P. squamosissimus* advertisement calls occur in dense choruses in November–December, the peak of the spawning season in low-water, and advertisement-like calls are also produced in July–August outside of the mating season (high-water). Males called from cages during the low but not high-water season, which confirms the caller’s identity and suggest greater spawning motivation during the low-water season. We suggest that the absence of cage calls outside of the spawning season reflects suboptimal conditions due to the high density of fish in the cages. At this site photoperiod is constant (LD 12∶12), temperature varies by about 3°C annually, and seasonal changes in water level, pH and conductivity may signal the onset of the mating season. In August–September, female GSI was at a low point for the year in the Amazon estuary [25] (see their Fig. 6), and for the fish in this study July–August females had undeveloped gonads (unpublished data). However, approximately 50% of males from a Northeast Brazilian reservoir are mature in August [26], which suggests a smaller number of potentially-courting males as reflected in sparser winter choruses. We therefore hypothesize that the seasonal range in androgen levels would vary less in the Amazon than in temperate latitudes where calling would stop outside of the mating season, as demonstated in weakfish *Cynoscion regalis* [1]. A parallel example occurs in the oyster toadfish *Opsanus tau*: most boatwhistles, an advertisement call, are produced in May through July, but after a silent period in August there is a small secondary calling season in the fall when a few short-duration low-frequency calls are produced [27]. These late-season boatwhistles occur simultaneously with a parallel increase in androgen levels [28].

Fewer shorter calls with fewer pulses per burst and longer periods between pulses (pulse period) in winter *P. squamosissimus* calls suggest that steroid levels affect the output of central pattern generators that control the timing of motor neuron firing. However the similarity in cycle period in low- and high-water bursts as well as in disturbance calls from both seasons suggests these factors don’t affect sonic muscle twitch duration. Similarly differences in peak frequency and cycle duration of disturbance and wild calls appear to relate to CNS output rather than muscle twitch parameters. Disturbance calls have one intense cycle followed by decay from a rapidly damped swimbladder. This waveform is likely caused by a single twitch as demonstrated by electromyography in weakfish *Cynoscion regalis* [14]. Based on the waveform and the number of cycles, individual pulses in advertisement bursts are likely caused by multiple contractions, i.e. the time period between successive muscle contractions rather than the time for a complete contraction-relaxation cycle determines peak frequency [29].

Disturbance calls in this study were recorded in air, and advertisement calls in water. Fine et al. [30] examined calls from the same individuals in Atlantic croaker recorded in both air and water and found peak frequencies were identical in both media although sharpness of tuning and damping were affected. Peak frequency is determined by the time for the swimbladder to be compressed and expand (e.g. sonic muscle contraction and relaxation time during a twitch), which occurs internally and is not affected by the medium. Therefore differences between peak frequencies between disturbance and advertisement calls in this study should relate to differences in muscle contraction between the calls and not the medium. The additional peaks at intervals of about 40 Hz that ride on the spectrum in both aerial and underwater recordings are similar to peaks found in an Australian sciaenid, *Argyrosomus japonicus* [6].

The advertisement call of *P. squamosissimus*, starting with individual and paired pulses and continuing as a series of long bursts, is complex for the family; sciaenids typically produce a series of individual pulses although pulse bursts have been recorded in other sciaenids. For instance nine Taiwanese species produce different pulse durations and patterns [4]. The speckled sea trout *Cynoscion nebulosus* produces a couple of different call types in choruses [17], but they do not form a complex pattern. The black drum *Pogonius chromis*, with atypical intrinsic muscles, produces long-duration tonal calls, but they are also highly repetitious [9], [10]. Interestingly disturbance calls in the white-mouth croaker *Micropogonius furnieri* [16] and the weakfish *Cynoscion regalis* [14], [32] and *Johnius macrorhynus* [31] produce relatively simple advertisement calls with several pulses and disturbance calls with a longer series of rapid pulses; contrary to what occurs in *P. squamissimus*, which produces more simple disturbance calls but more complex advertisement calls.

Disturbance calls in small *P. squamissimus* (13.0–19.0 cm SL) were too weak to hear although vibrations were felt when holding the fish. Sonic muscles in sciaenids develop post-embryonically coincident with sexual maturation from undeveloped gonads in juveniles to identifiable testes and ovaries [11]. *P. squamissimus* in the central Amazon mature between 19.0 and 20.5 cm [33], the size range when sounds were first heard. Therefore the developing muscles, which grow from an aponeurosis on the dorsal swimbladder to their origin on the ventral midline in spot, croaker and weakfish [8], [11] may contract before they are fully developed, which to our knowledge has not been reported before. Additionally sciaenid sonic muscles undergo a yearly hypertrophy-atrophy cycle that correlates with androgen levels so that muscles are prepared for extensive calling during the reproductive season [2], [13], [14]. We observed more developed muscles during the low-water mating period in *P. squamissimus* (Fig. 2). Therefore muscle state is unlikely to affect twitch time based on similar cycle periods of disturbance calls in and out of the mating season. However, muscle development likely affects sound amplitude and fatigue resistance. Devincenti et al [34] found sonic muscles in post spawning white mouth croaker *Micropogonias furnieri* in Uruguay were negative for lipids and succinic dehydrogenase, a marker for mitochondria. Furthermore, they were only weakly positive for glycogen, suggesting lower fatigue resistance outside of the mating season [35], and sonic muscles in *Cynoscion regalis*, increase lipid, glycogen and protein during the mating season [14].

Despite a constant photoperiod and minimal annual cycle in temperature, sonic muscles in *Plagioscion squamosissimus* undergo a seasonal hypertrophy-atrophy cycle as in temperate sciaenids. However, advertisement calls are produced in both seasons although call bursts are longer and more frequent during the mating season. Equatorial fishes must rely on cues that are different from temperate species, and future work will be necessary to determine the specific nature of the cues for calling and their variation in different species.

## Supporting Information

File S1 ***Plagioscion squamosissimus*** **: chorus during the mating season.**
(WAV)

File S2 ***Plagioscion. squamosissimus*** **: individual advertisement call during the low-water mating season.**
(WAV)

File S3 ***Plagioscion. squamosissimus*** **: chorus of advertisement-like calls during the high-water non-mating season.**
(WAV)
